# Enhanced Forward Scattering of Ellipsoidal Dielectric Nanoparticles

**DOI:** 10.1186/s11671-016-1794-x

**Published:** 2017-01-19

**Authors:** Zhonghua Wang, Ning An, Fei Shen, Hongping Zhou, Yongxuan Sun, Zhaoneng Jiang, Yanhua Han, Yan Li, Zhongyi Guo

**Affiliations:** 1 School of Information Science and Technology, University of Science and Technology of China, Hefei, 230026 China; 2 School of Computer and Information, Hefei University of Technology, Hefei, 230009 China; 3 Department of Optoelectronics Science, Harbin Institute of Technology at Weihai, Weihai, 264209 China; 4 State Key Laboratory of Millimeter Waves, Southeast University, Nanjing, 210096 China

**Keywords:** Dielectric nanoparticle, Ellipsoidal nanoparticle, Forward scattering, Electric and magnetic dipolar resonances

## Abstract

Dielectric nanoparticles can demonstrate a strong forward scattering at visible and near-infrared wavelengths due to the interaction of optically induced electric and magnetic dipolar resonances. For a spherical nanoparticle, the first Kerker’s condition within dipole approximation can be realized, where backward scattering can reach zero. However, for this type of dielectric sphere, maximum forward scattering without backward scattering cannot be realized by modulating the refractive index and particle size of this nanoparticle. In this paper, we have demonstrated that a larger directional forward scattering than the traditional spherical nanoparticle can be obtained by using the ellipsoidal nanoparticle, due to the overlapping electric and magnetic dipolar modes. For the oblate ellipsoid with a determined refractive index, there is an optimum shape for generating the suppressed backward scattering along with the enhanced forward scattering at the resonant wavelength, where the electric and magnetic dipolar modes overlap with each other. For the prolate ellipsoid, there also exist the overlapping electric and magnetic dipolar modes at the resonant wavelength of total scattering, which have much higher forward scattering than those for both oblate ellipsoid and sphere, due to the existence of the higher multipolar modes. Furthermore, we have also demonstrated the realization of the dimensional tailoring in order to make the strong forward scattering shift to the desired wavelength.

## Background

Electromagnetic waves scattered by nanoparticles has gained great attention because of its immense applications, including optical communications [1, 2], optical manipulations [3, 4], material science [5, 6], and so on. In general, scattering ordinarily relies on shape, size, and composition of nanoparticles. Due to its remarkable electric and magnetic resonances at optical frequencies, the scattering of dielectric nanoparticles has attracted lots of renewed interest in the last few years. The interactions between the electric and magnetic modes allow to fulfill certain conditions, which can generate directional scattering [7–14]. Such certain condition was called Kerker’s condition which was first proposed with small magnetodielectric spheres in 1983 [7], including the first Kerker’s condition for zero backward scattering (BS) and the second Kerker’s condition for minimum forward scattering (FS). In the middle of these two particular scattering properties, suppressed backward scattering and enhanced directional forward scattering are typically of more practical application, such as in optical antennas [15–17], plasmon-enhanced photovoltaics [18, 19], and other devices based on optically induced “negative forces” [20].

The proposed interactions between the electric and magnetic modes caused by dielectric nanoparticles could manipulate angular distributions of the scattering more flexibly than the pure electric-response-based method, which is usually driven by complex structures [21, 22]. Nevertheless, the zero backward scattering condition for spherical dielectric particles can only be fulfilled in the longer wavelength region than the magnetic dipole resonant wavelength. Moreover, such spherical nanoparticles are governed by only one geometrical parameter and thus do not allow for the spectral-shift of the resonant-position for the electric and magnetic dipoles. Recently, it was shown that in silicon nanodisks with the aspect ratio about 1:2, electric and magnetic dipolar resonances can be overlapped to utilize the spectral-shift of the resonant-position [23], providing a strong FS and near zero BS at the scattering resonance wavelength.

In this paper, we give a general discussion on the light scattering by the spherical and ellipsoidal dielectric nanoparticles. Firstly, with the method of Mie theory, it is shown that the scattered field for a plane wave illuminating the dielectric sphere can be decomposed into a series of electric and magnetic multipolar modes in free space. From the Mie decompositions of the scattered field, we can obtain the condition of the zero backward scattering. However, maximum forward scattering without backward scattering occurs away from the resonant wavelength of total scattering. Then, we present numerical parametric researches for the oblate ellipsoid and prolate ellipsoid by using finite element method and multipole decomposition based on electromagnetic multipole theory, which particularly demonstrate the possibility of suppressed backward scattering and enhanced directional forward scattering. For oblate ellipsoid, we can find an optimum aspect ratio usually, with near zero backward scattering and enhanced forward scattering at the resonant wavelength of total scattering, due to the overlapping of the electric and magnetic dipole resonances. For prolate ellipsoid, we can also find the overlapping of the electric and magnetic dipole resonances similar to that of oblate ellipsoid, where the forward scattering can be enhanced more strongly while the backward scattering is not zero due to the existence of the higher-order multipolar modes. Finally, we also provide an easy way to tune the strong forward scattering to the desired wavelength.

## Methods

Scattering properties of a spherical dielectric nanoparticle in free space have been solved analytically based on Mie theory [24]. The spherical dielectric nanoparticle has a radius of *r* and the refractive index of *n*. The incident plane wave is assumed to propagate along *x*-direction and polarized along the *z*-direction. The scattered field can be decomposed into electric and magnetic multipolar modes (the so-called Mie’s expansion), in which the Mie coefficients can be expressed as ${a}_m=\left|{a}_m\right|{e}^{i{\gamma}_m}$ and ${b}_m=\left|{b}_m\right|{e}^{i{\delta}_m}$, respectively (*γ*_*m*_ and *δ*_*m*_ are the corresponding phase of the electric and magnetic responses). And the total scattering efficiency *Q*_*sca*_ can be defined as the ratio of the scattering cross section divided by the cross section (*πr* ^2^) of the particle [24]:

1 $$ {Q}_{sca}=\frac{2}{k^2{r}^2}{\displaystyle {\sum}_{m=1}^{\infty}\left(2m+1\right)\left({\left|{a}_m\right|}^2+{\left|{b}_m\right|}^2\right)}, $$

where *k* is the wavenumber. Considering that *a*_*m*_ and *b*_*m*_ correspond to the *m*th order electric and magnetic multipoles, respectively (e.g., *a*_1_ and *b*_1_ correspond to electric dipole and magnetic dipole, respectively), then the *Q*_*sca*_ contributed from them can be expressed respectively as follows:

2 $$ {Q}_{am}=\frac{2}{k^2{r}^2}\left(2m+1\right){\left|{a}_m\right|}^2,\ {Q}_{bm}=\frac{2}{k^2{r}^2}\left(2m+1\right){\left|{b}_m\right|}^2. $$

Furthermore, The BS efficiency (*Q*_*bs*_) and the FS efficiency (*Q*_*fs*_), which respectively correspond to the scattering efficiency at the backward and forward direction [24], can be expressed as follows:

3 $$ {Q}_{bs}=\frac{1}{k^2{r}^2}{\left|{\displaystyle {\sum}_{m=1}^{\infty }{\left(-1\right)}^m\left(2m+1\right)\left({a}_m-{b}_m\right)}\right|}^2 $$

4 $$ {Q}_{fs}=\frac{1}{k^2{r}^2}{\left|{\displaystyle {\sum}_{m=1}^{\infty}\left(2m+1\right)\left({a}_m+{b}_m\right)}\right|}^2 $$

## Results and discussions

**Fig. 1:**
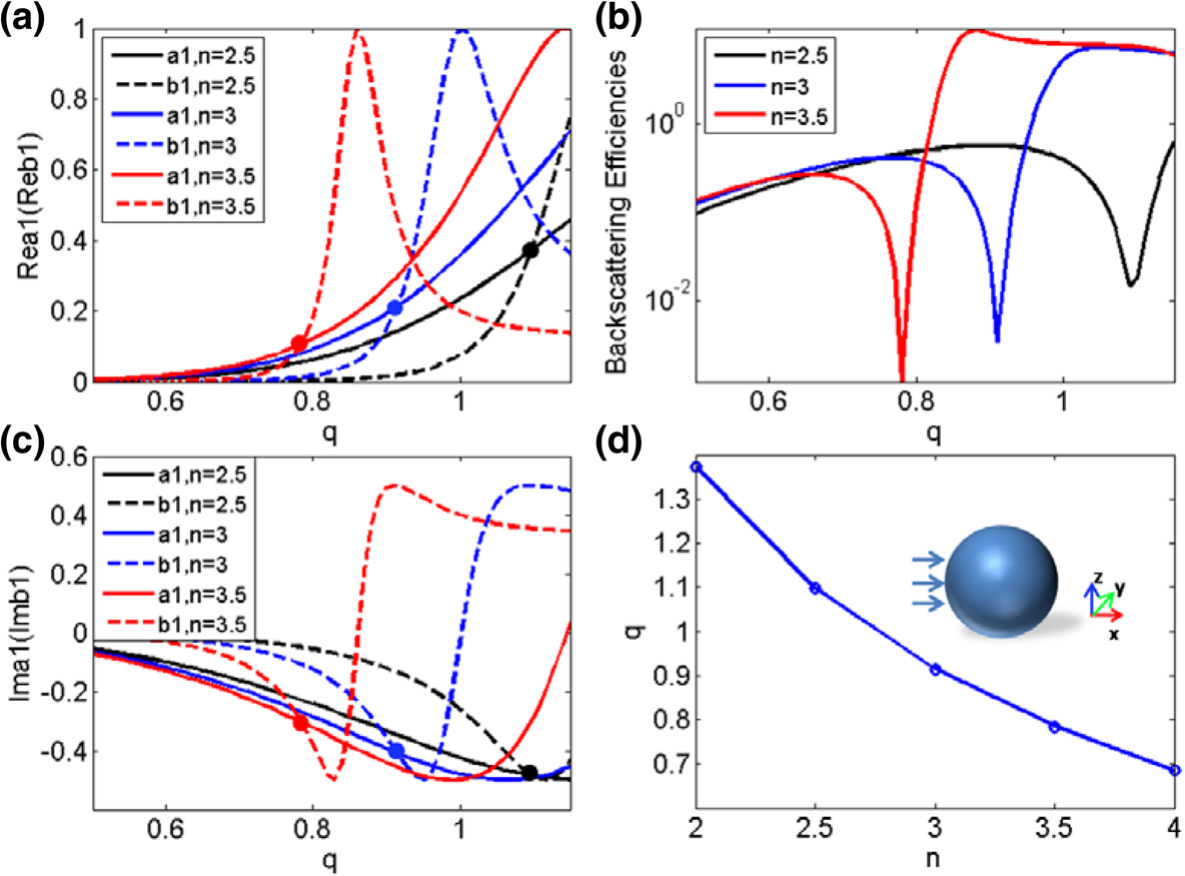
The real (**a**) and imaginary parts (**c**) of electric dipole coefficient *a*_1_ and magnetic dipole coefficient *b*_1_ for spherical dielectric nanoparticles with three different values of refractive index *n* = 2.5, 3, and 3.5 respectively. The *filled black*, *blue*, and *red circles* show Re*a*_1_ = Re*b*_1_ and Im*a*_1_ = Im*b*_1_ corresponding to *n* = 2.5, 3, and 3.5 respectively. **b** Backward scattering efficiency (in the logarithmic scale) as a function of *q* with three above refractive indexes. **d** Trajectory of minimum backward efficiency on the plane of parameters *n* and *q*

Firstly, we characterize the condition of the first Kerker’s condition within dipole approximation, i.e., *a*_1_ = *b*_1_, for a dielectric sphere with *r* = 0.24μm. This is due to the fact the lowest order dipolar modes are easiest to excite, and thus to a certain degree, these two modes will dominantly determine the scattering pattern [7–12, 23, 25–28]. Figure 1a, c shows the real and imaginary parts of *a*_1_ and *b*_1_ as a function of *q* (*q* is particle size parameter), for three different values of refractive index *n* = 2.5, 3, and 3.5. The conditions *a*_1_ = *b*_1_ corresponding to *n* = 2.5, 3, and 3.5 are marked by filled black, blue, and red circles in Fig. 1a, c. Satisfying the condition for both real and imaginary parts will lead to pronounced minima in the backward scattering efficiencies as depicted in Fig. 1b. In this case, the zero backscattering occurs when the electric and magnetic dipoles have the same strength and oscillated phase. Since the natures of these dipolar radiations, the forward scattering becomes constructive, while the backward scattering is reduced to near zero. Figure 1d presents the trajectory of minimum backward scattering efficiency on the *q*, *n* parameters’ plane. And according to the condition *a*_1_ = *b*_1_, we can conclude that the trajectory of minimum backward scattering efficiency can be well depicted by *qn* = 2*πrn*/*λ* ≈ 2.75 (*λ* is the incident wavelength) [9, 25]. The inset shown in Fig. 1d depicts the schematic diagram of single spherical nanoparticle and associated coordinate system in the simulations. In this work, we just consider electromagnetic scattering of nanoparticles in the vacuum.

**Fig. 2:**
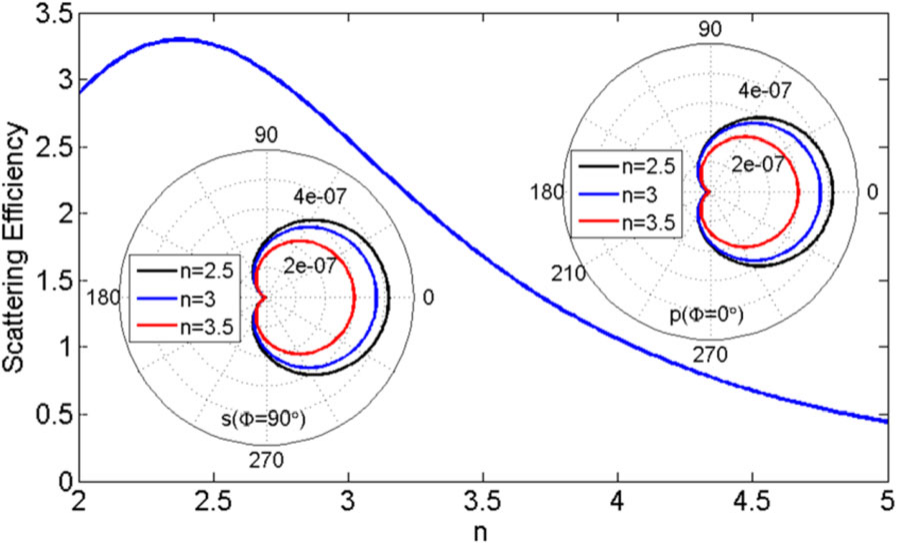
The total scattering efficiency of a spherical dielectric nanoparticle as a function of the refractive index *n* under first Kerker’s condition within dipole approximation. In the *insets*, the two-dimension scattering patterns are further calculated for spherical dielectric nanoparticles of *r* = 0.24 μm versus *n* = 2.5, 3, and 3.5 corresponding to λ = 1.38, 1.65, and 1.93 μm

In Fig. 2, we show the total scattering efficiency for a sphere with different refractive index, where the first Kerker’s condition within dipole approximation holds. Here, our aim is to find the optimal refractive index of the sphere with the largest scattering efficiency under the first Kerker’s condition within dipole approximation, in which we can find that the spherical nanoparticle with refractive index around 2.5 has the largest scattering efficiency. For example, there are three real materials possessing a refractive index within this range [29], such as diamond, titanium dioxide, and strontium titanate. The two-dimension scattering patterns are further calculated for spherical dielectric nanoparticles of *r* = 0.24μm versus *n* = 2.5, 3, and 3.5 corresponding to *λ* = 1.38, 1.65, and 1.93 μm, respectively, and displayed in the insets. Right inset presents the scattering pattern of *xz*-plane corresponding to the *p*-polarized component (azimuthal angle *ϕ* = 0*°*) and the left one presents the scattering pattern of *xy*-plane corresponding to the *s*-polarized component (*ϕ* = 90*°*). It is found that the backward scatterings are almost completely suppressed and scattered energies are radiated into the forward direction, which shows that the suppressing backward scattering and the largest forward scattering can be achieved with refractive index around 2.5 under the first Kerker’s condition within dipole approximation.

**Fig. 3:**
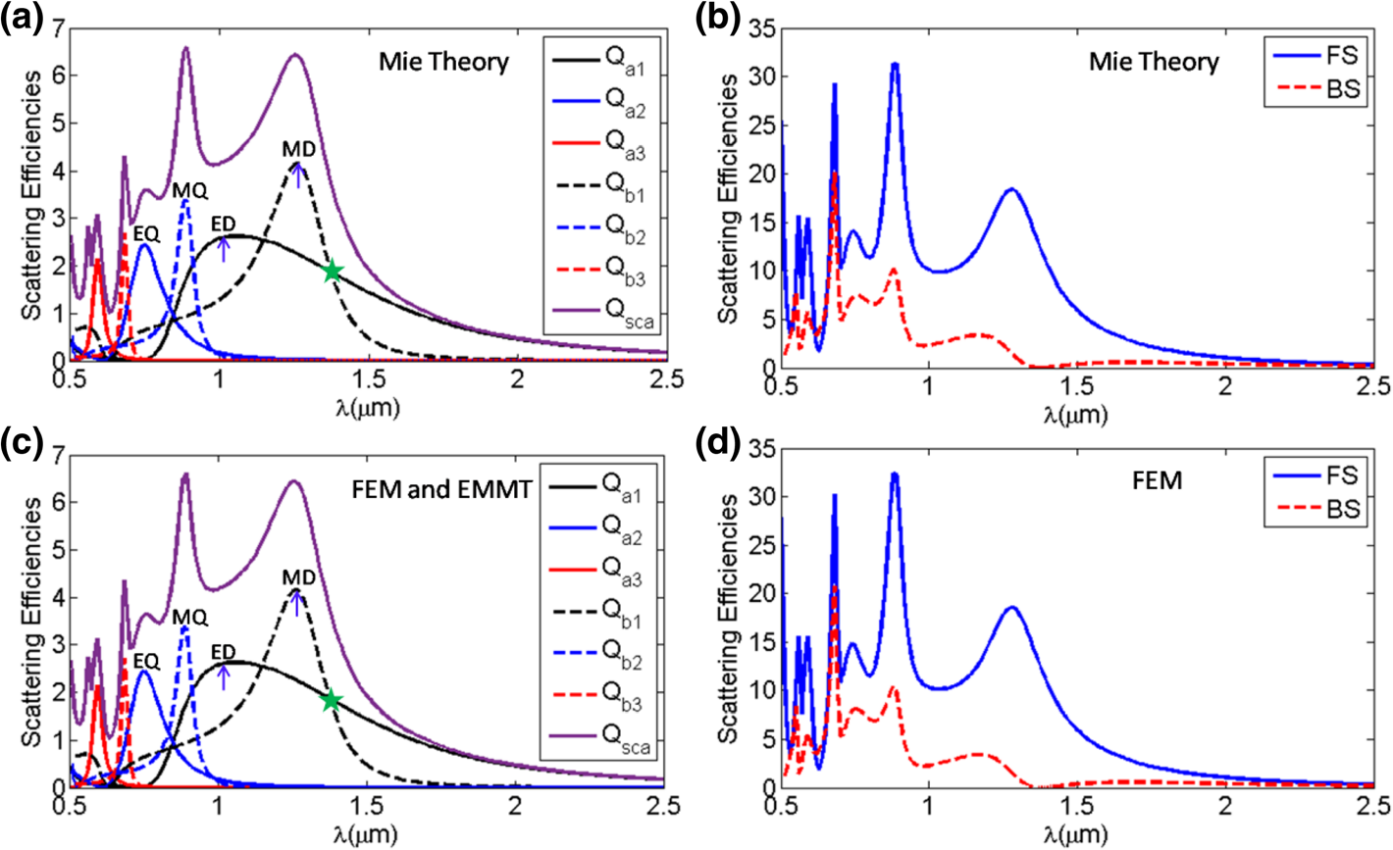
The total with corresponding multipole contributions (**a**), forward and backward (**b**) scattering efficiencies of spherical dielectric nanoparticle with *r* = 0.24 μm and *n* = 2.5 by using Mie theory. The total with corresponding multipole contributions (**c**), forward and backward (**d**) scattering efficiencies of spherical dielectric nanoparticle with *r* = 0.24 μm and *n* = 2.5 by using FEM and EMMT. *Q*_*a*1_ (ED) and *Q*_*b*1_ (MD) represent the electric and magnetic dipole responses, respectively. *Q*_*a*2_ (EQ) and *Q*_*b*2_ (MQ) represent the electric and magnetic quadrupole responses, respectively. *Q*_*a*3_ and *Q*_*b*3_ represent the electric and magnetic octupole responses, respectively. The *arrows* show the locations of corresponding multipole contributions

From above, we can conclude that the largest scattering efficiency for spherical particles occurs at the refractive index around 2.5, where the first Kerker’s condition can be satisfied within dipole approximation. In the following, we will focus on the scattering properties of the spherical particles with *r* = 0.24 μm, *n* = 2.5 by using Mie theory (Fig. 3a, b) and finite element method (FEM) (Fig. 3c, d) respectively, which show FEM is an effective way because it matches well with the Mie theory. Meanwhile, in order to gain further insight into the origin of directional scattering properties, the corresponding electric and magnetic multipole contributions can be calculated from both Eqs. (2) (Fig. 3a) and electromagnetic multipole theory (EMMT) (Fig. 3c) [30]. It is clearly seen that the EMMT results agree well with the ones calculated by Mie theory. Therefore, we would use FEM and EMMT for obtaining the scattering of the spheroid in the following, which can calculate the scattering properties and corresponding electric and magnetic multipole contributions. At the wavelength *λ* = 1.38 μm marked as green start as shown in Fig. 3a, c, where the first Kerker’s condition within dipole approximation (*a*_1_ = *b*_1_) is satisfied as depicted in Fig. 1, the backward scattering is almost zero but the total and forward scattering takes place at the tail of magnetic dipole resonance. This means that for a spherical particle, whatever the particle parameters are, it is not possible to obtain much larger forward scattering by fulfilling the first Kerker’s condition within dipole approximation for the minimum backward scattering. From formula (4), the condition leads to that the forward scattering is proportional to the value of *n* ^2^|*a*_1_|^2^. However, the values of |*a*_1_| or |*b*_1_| at the first Kerker’s condition within dipole approximation are quite small, below 0.6, as seen in Fig. 1a, c. Therefore, if the electric and magnetic dipolar modes could be overlapped, both the total and forward scattering would be enhanced.

To achieve a relatively strong forward scattering, one of the possibilities is to use metallic-dielectric core-shell nanoparticles [14, 26–28]. In the following, we will demonstrate that it can also be realized by changing the particle’s shape, e.g., using oblate ellipsoid or prolate ellipsoid instead of the sphere. As exhibited in Ref. [23], by using a nanodisk instead of sphere with an aspect ratio near 1:2, they could make the electric and magnetic dipole resonances overlap and make the minimized backward scattering approach to the wavelength of scattering resonance. As an effective way to reach a strong forward scattering, we will talk about it for both oblate ellipsoid and prolate ellipsoid.

**Fig. 4:**
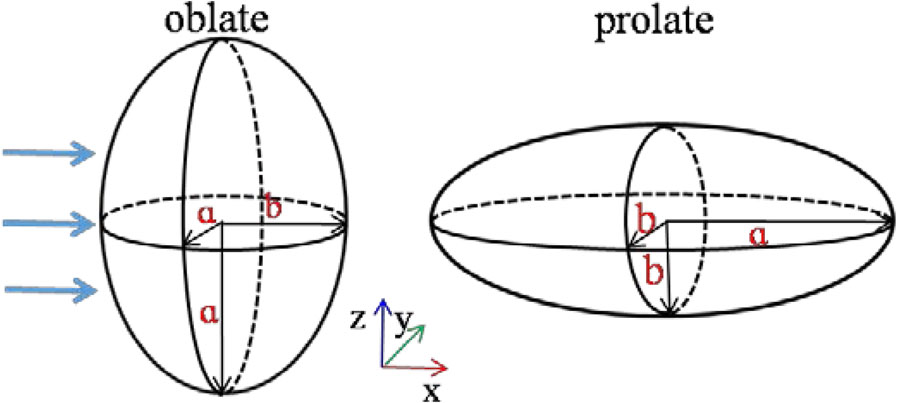
Schematic diagrams of the used oblate ellipsoid, prolate ellipsoid, and associated coordinate system in the simulations

A spheroid (ellipsoid of revolution) is obtained by the rotation of an ellipse around its minor axis (oblate ellipsoid) or its major axis (prolate ellipsoid). The aspect ratio defines as the ratio of the major semiaxis (a) to the minor semiaxis (b), which describes the particle shape vary from a sphere (*a*/*b* = 1) to a disk for oblate ellipsoid or a needle for prolate ellipsoid (*a*/*b* ≠ 1). Also, we define the particle size parameter as *q*_*v*_ = 2*πr*_*v*_/*λ* (*r*_*v*_ is the radius of sphere which has the same volume to that of the spheroid). Schematic diagrams of oblate ellipsoid and prolate ellipsoid and associated coordinate system under study are shown in Fig. 4. In this work, the incident plane wave is assumed to propagate along *x*-direction (minor axis for oblate ellipsoid or major axis for prolate ellipsoid) and polarized along *z*-direction. The following numerical calculations are performed using FEM and EMMT for the spheroid.

**Fig. 5:**
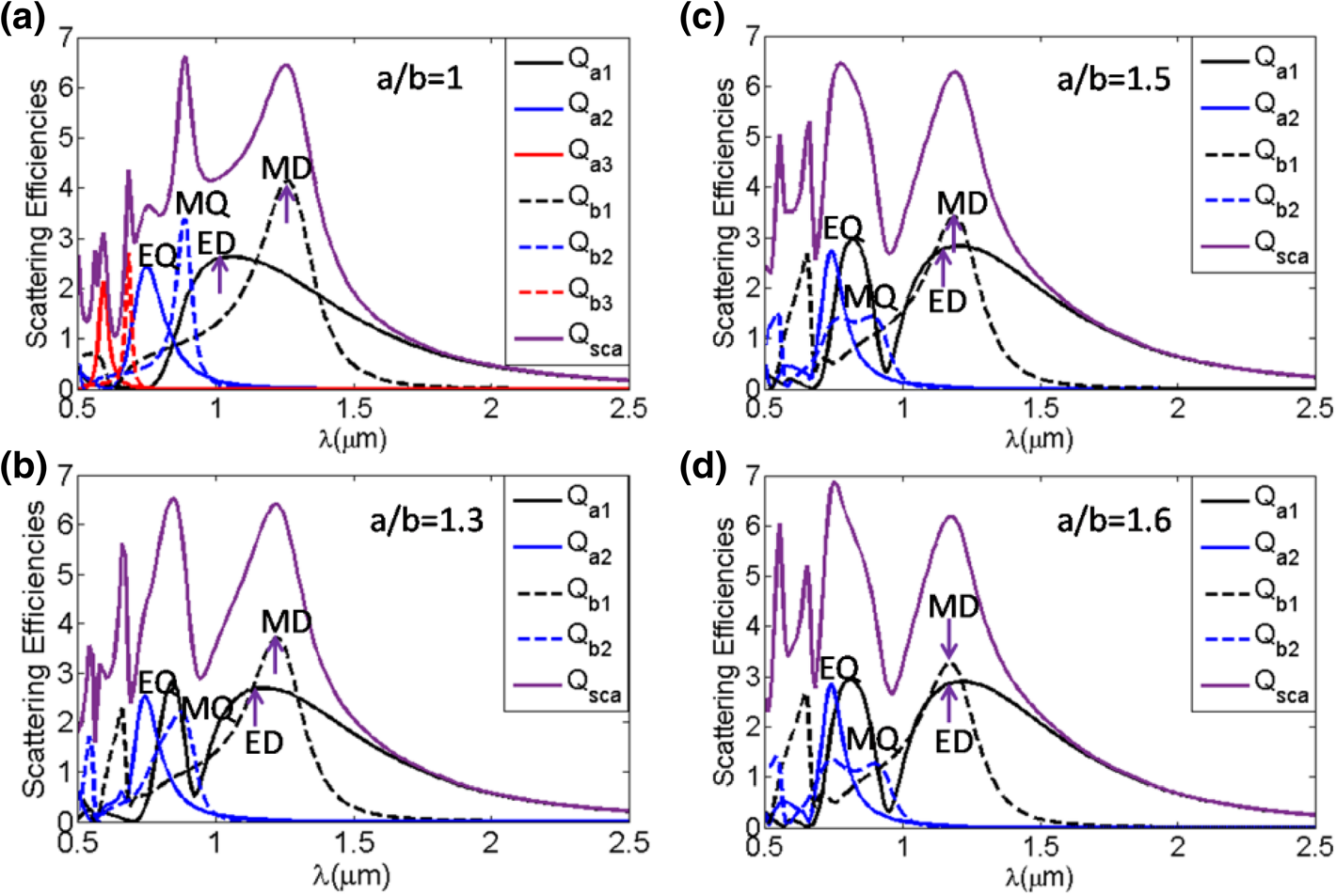
The total scattering efficiency with corresponding multipole contributions of oblate ellipsoid nanoparticles for different aspect ratio a/b, ranging from 1 to 1.6. *Q*_*a*1_ (ED) and *Q*_*b*1_ (MD) represent the electric and magnetic dipole resonances, respectively. *Q*_*a*2_ (EQ) and *Q*_*b*2_ (MQ) represent the electric and magnetic quadrupole resonances, respectively. *Q*_*a*3_ and *Q*_*b*3_ represent the electric and magnetic octupole resonances, respectively. The *arrows* show the locations of corresponding multipole contributions

Now the performance of the oblate ellipsoid, which considers as *r*_*v*_ = 0.24μm and *n* = 2.5, is illustrated in Fig. 5. We depict the total scattering characteristic as a function of the incident wavelength, as shown in Fig. 5, and the corresponding electric and magnetic multipole contributions to the total scattering efficiency are also shown in Fig. 5. It is evident that some shifts in the position of the resonance wavelengths due to deformation of the sphere, which show the tuned dynamics of the overlapping between the electric and magnetic dipole resonances for the oblate ellipsoid nanoparticle with refractive index *n* = 2.5. But for the spherical particle, electric and magnetic dipolar resonances are well separated. From Fig. 5, it is seen that the electric and magnetic dipolar resonances approach each other gradually with increasing a/b, which can allow to obtain minimum backward scattering at the resonance of total scattering. It is noted that total scattering efficiency of oblate ellipsoid with *a*/*b* = 1.6, where the electric and magnetic dipolar modes are overlapped, occurs at the resonance of total scattering.

**Fig. 6:**
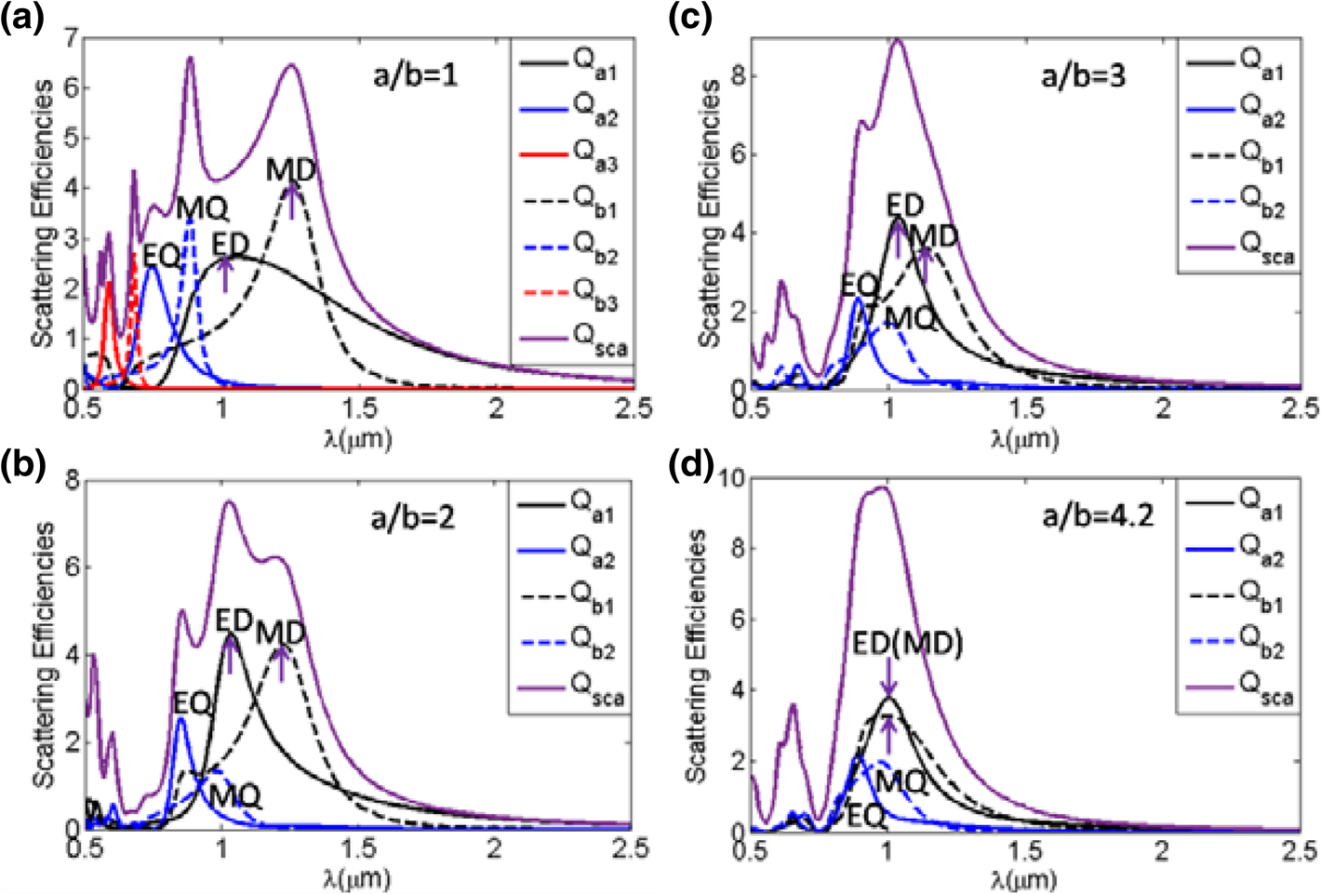
The total scattering efficiency with corresponding multipole contributions of prolate ellipsoid nanoparticles for different aspect ratio a/b, ranging from 1 to 4.2. *Q*_*a*1_ (ED) and *Q*_*b*1_ (MD) represent the electric and magnetic dipole responses, respectively. *Q*_*a*2_ (EQ) and *Q*_*b*2_ (MQ) represent the electric and magnetic quadrupole responses, respectively. *Q*_*a*3_ and *Q*_*b*3_ represent the electric and magnetic octupole responses, respectively. The *arrows* show the locations of corresponding multipole contributions

Next, we will discuss the performances of the prolate ellipsoids. Alike the case of oblate ellipsoid, we focus on the total scattering and the corresponding multipole contributions with *r*_*v*_ = 0.24 μm and *n* = 2.5 versus different aspect ratios, as shown in Fig. 6. It is clearly shown that the total scattering have some shift in the position of the resonance wavelengths due to deformation of the sphere. It is also seen that the electric and magnetic dipolar resonances approach each other gradually with increasing a/b, which can allow obtaining minimum backward scattering at the resonance of total scattering. It is noted that total scattering efficiency of prolate ellipsoid with *a*/*b* = 4.2, where the electric and magnetic dipolar modes are overlapped, occurs at the resonance of total scattering. In this particle, we can realize a larger total scattering than the ones of sphere and oblate ellipsoid at the overlapping of the electric and magnetic dipolar resonances. It is due to the existence of the higher multipolar modes at the overlapping of the electric and magnetic dipolar resonances as shown in Fig. 6, which could enhance the scattering more strongly.

**Fig. 7:**
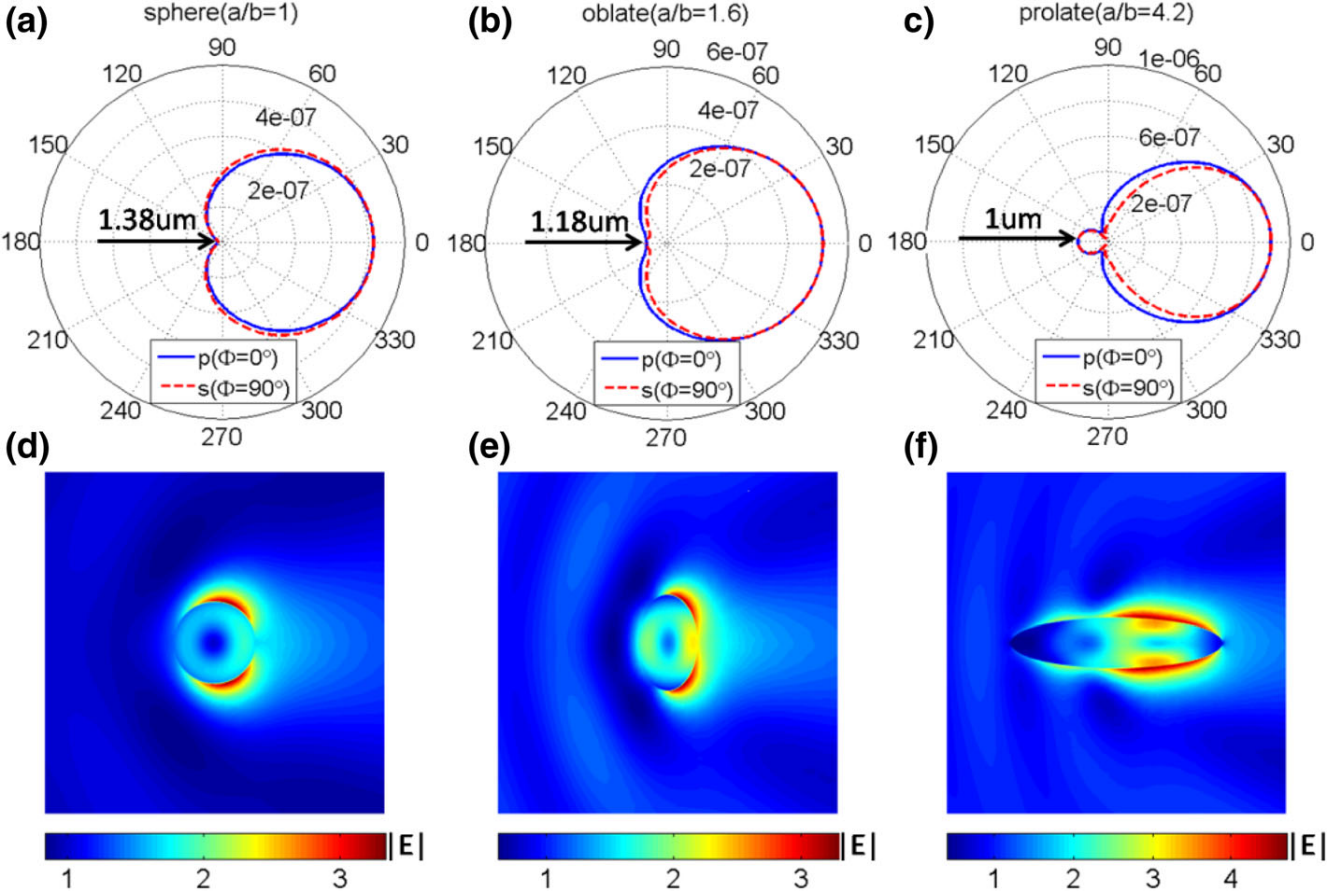
The two-dimension scattering patterns for sphere (*a*/*b* = 1) (**a**), oblate ellipsoid (*a*/*b* = 1.6) (**b**), and prolate ellipsoid (*a*/*b* = 4.2) (**c**) dielectric nanoparticles with same *n* = 2.5, consisting of both *p*-polarized and *s*-polarized components. The corresponding electric field distributions around the nanoparticles of sphere (*a*/*b* = 1) (**d**), oblate ellipsoid (*a*/*b* = 1.6) (**e**), and prolate ellipsoid (*a*/*b* = 4.2) (**f**)

Typical two-dimension scattering patterns are further illustrated in Fig. 7a–c for sphere (*a*/*b* = 1), oblate ellipsoid (*a*/*b* = 1.6), and prolate ellipsoid (*a*/*b* = 4.2) corresponding to *λ* = 1.38, 1.18, and 1 μm with the same *n* = 2.5. And the arrows in Fig. 7a–c denote the incident wave direction. It is found that the scattering in the backward direction is suppressed at above situations. It is also seen that only a small part of the scattered energy is radiated into the backward hemisphere for both *p*-polarized and *s*-polarized components. Given the comparison in Fig. 7a, b, it is clearly seen that the forward scattering can be enhanced strongly, while the backward scattering is suppressed. For prolate ellipsoid (*a*/*b* = 4.2), the forward scattering is much more strong than those for both sphere and oblate ellipsoid. And the backward scattering is not zero but much smaller than the forward one. It is due to the existence of the higher multipolar modes, which can be seen in Fig. 6. Moreover, the scattered energy is mainly radiated into the forward hemisphere. Furthermore, the corresponding electric field distributions (*xz*-plane), around the nanoparticles of sphere (*a*/*b* = 1), oblate ellipsoid (*a*/*b* = 1.6), and prolate ellipsoid (*a*/*b* = 4.2), have been shown in Fig. 7d–f. It can be seen that the electric field is suppressed in the backward direction and the values of the electric field are relatively large in the forward direction, which agree well with the far scattering patterns. These figures confirm that the enhanced forward scattering combined with suppressed backward scattering can be obtained by deformation of the sphere.

**Fig. 8:**
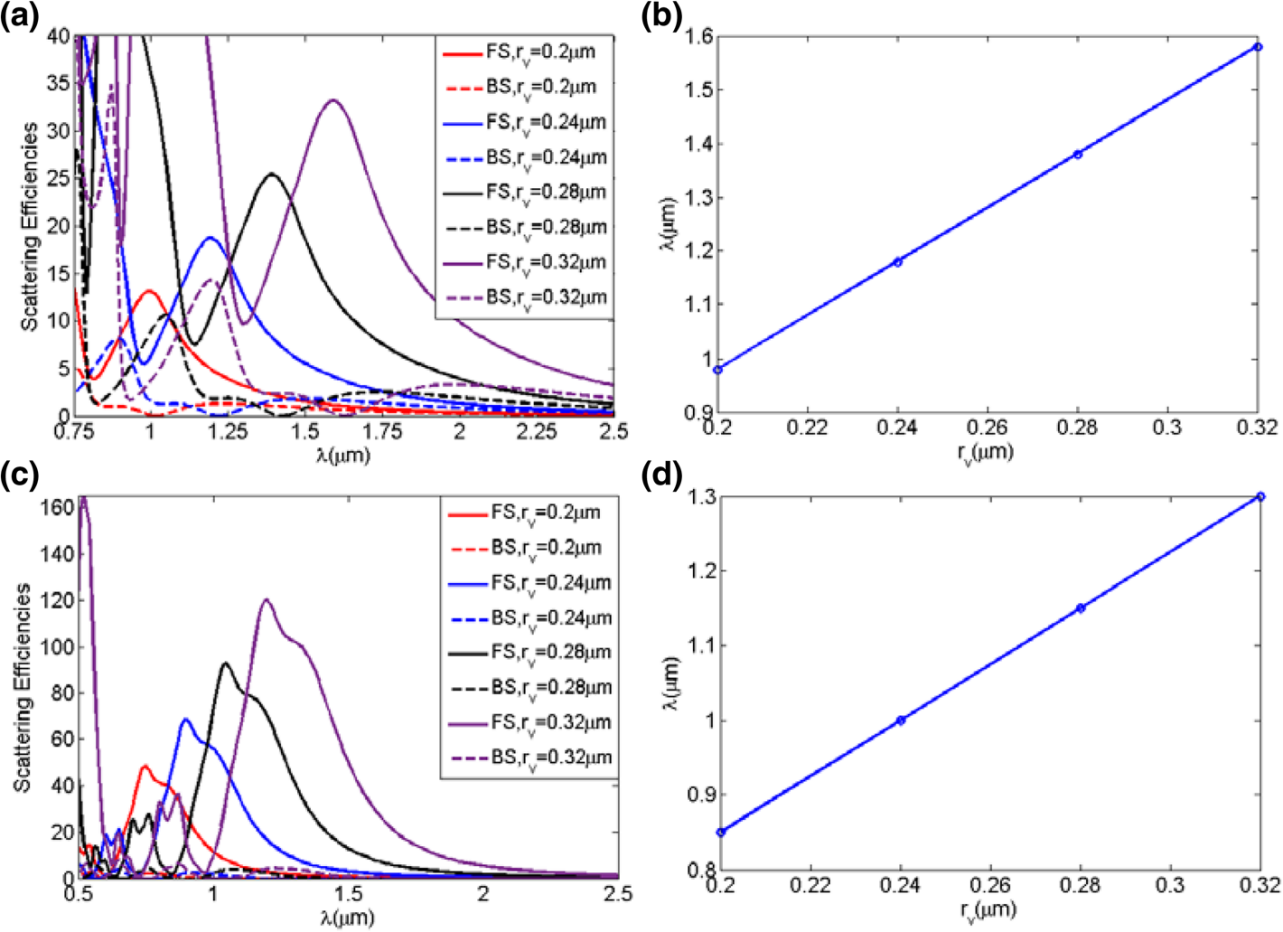
The backward and forward scattering efficiencies for oblate ellipsoids (**a**) and prolate ellipsoids (**c**) versus the incident wavelength for different particle size from *r*_*v*_ = 0.32 μm to *r*_*v*_ = 0.20 μm. **b** and **d** show the trajectories of wavelength with a/b, which has suppressed backward scattering and enhanced forward scattering for oblate ellipsoids and prolate ellipsoids, respectively

In general, the scattering performances of the nanoparticles are dependent on the size parameters, which makes it possible to tune the suppressed backward scattering and enhanced forward scattering for the oblate ellipsoid and prolate ellipsoid to the desired wavelength by varying the geometrical parameters. In order to demonstrate the tuning mechanism further, the backward and forward scattering efficiencies for the oblate ellipsoids and prolate ellipsoids with four different size of *r*_*v*_ = 0.32, 0.28, 0.24, and 0.20 μm are displayed in Fig. 8a, c, respectively, as functions of the incident wavelength. Figure 8b, d shows the trajectories of the incident wavelength with *a*/*b*, which has suppressed backward scattering and enhanced forward scattering for oblate ellipsoids and prolate ellipsoids, respectively. Comparing all the case as shown in Fig. 8, it is obvious that the suppressed backward scattering and enhanced forward scattering gradually shift to the longer wavelength with increasing particle size for both oblate ellipsoids and prolate ellipsoids.

## Conclusions

In this paper, the scattering characteristics of the spherical and ellipsoidal dielectric nanoparticles have been investigated, in order to obtain enhanced total and forward scattering together with suppressed backward scattering. Ellipsoidal nanoparticles with different aspect ratios provide an effective method for obtaining the overlapped electric and magnetic dipole resonances. For both oblate ellipsoid and prolate ellipsoid, we could obtain strong asymmetric scattering of suppressed backward scattering and enhanced forward scattering at the resonance wavelength of total scattering, with the given value of refractive index *n* = 2.5. Moreover, we could obtain larger total and forward scattering in the situation of prolate ellipsoid than those for both oblate ellipsoid and sphere, at which the electric and magnetic dipolar modes are overlapped at the resonance wavelength of total scattering. Finally, we have performed the size parameter study of light scattering for ellipsoidal structures, which can move the suppressed backward scattering and enhanced forward scattering to the desired wavelength. Overall, we provide a flexible mean to gain enhanced total and forward scattering together with suppressed backward scattering. The above mentioned properties make the ellipsoidal dielectric nanoparticles have great potential applications in manipulating light at nanoscale, such as solar cell application, efficient directional optical nanoantennas, sensing, and so on.
